# Seabird meta-Population Viability Model (mPVA) methods

**DOI:** 10.1016/j.mex.2021.101599

**Published:** 2021-12-09

**Authors:** M. Tim Tinker, Kelly M. Zilliacus, Diana Ruiz, Bernie R. Tershy, Donald A. Croll

**Affiliations:** a EEB Department, University of California Santa Cruz, Santa Cruz, CA USA; b Conservation Action Lab, University of California Santa Cruz, Santa Cruz, CA USA; c Nhydra Ecological Consulting, Nova Scotia, Canada

**Keywords:** mPVA, meta-Population Viability Analysis, IUCN, International Union for Conservation of Nature, AFR, Age of first reproduction, K, Carrying capacity, AoO, Area of occupancy, MLE, Maximum likelihood estimation, MCMC, Markov chain Monte Carlo analysis, R, R computer language for statistical computing, JAGS, Just another Gibbs Sampler, SSD, Stable stage distribution, QEP, Quasi-extinction probability, QE, Quasi-extinction threshold, Conservation, Population model, Extinction risk, Bayesian hierarchical model

## Abstract

The seabird meta-population viability model (mPVA) uses a generalized approach to project abundance and quasi-extinction risk for 102 seabird species under various conservation scenarios. The mPVA is a stage-structured projection matrix that tracks abundance of multiple populations linked by dispersal, accounting for breeding island characteristics and spatial distribution. Data are derived from published studies, grey literature, and expert review (with over 500 contributions). Invasive species impacts were generalized to stage-specific vital rates by fitting a Bayesian state-space model to trend data from Islands where invasive removals had occurred, while accounting for characteristics of seabird biology, breeding islands and invasive species. Survival rates were estimated using a competing hazards formulation to account for impacts of multiple threats, while also allowing for environmental and demographic stochasticity, density dependence and parameter uncertainty.

• The mPVA provides resource managers with a tool to quantitatively assess potential benefits of alternative management actions, for multiple species
• The mPVA compares projected abundance and quasi-extinction risk under current conditions (no intervention) and various conservation scenarios, including removal of invasive species from specified breeding islands, translocation or reintroduction of individuals to an island of specified location and size, and at-sea mortality amelioration via reduction in annual at-sea deaths

## Graphical abstract

**Figure:**
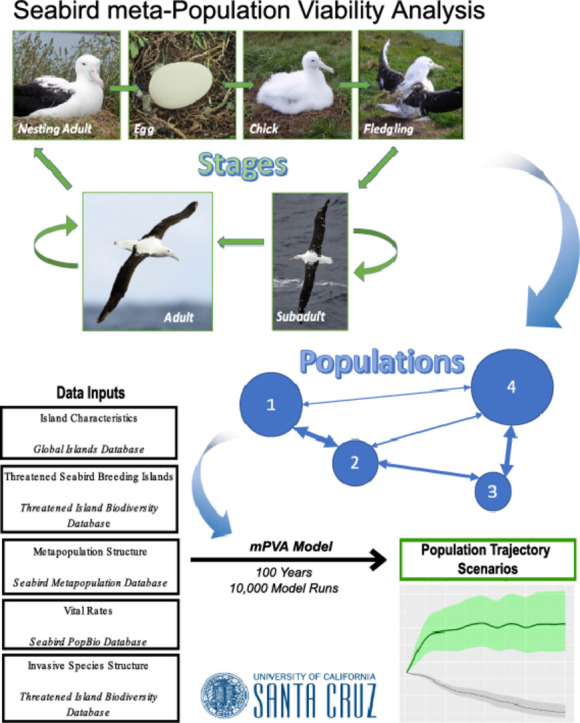

Specifications table

**Table:** 

Subject Area:	Environmental Science
More specific subject area:	Population Modeling
Method name:	Meta-Population Viability Model
Name and reference of original method:	[8]. Matrix population models: construction, analysis, and interpretation. 2nd ed edition. Sinauer Associates, Sunderland, MA..
Resource availability:	NA

## Overview

Mechanistic models of wildlife population dynamics have long been important tools for resource managers and conservation biologists [12]. A key advantage of using process-based models that include spatial and demographic structure is that the impacts of many threats (e.g., invasive species, fishing by-catch) are both spatially explicit and stage-specific, and thus the conservation benefits of mitigation efforts (e.g., removal of invasive species, fishing regulations) can be best-evaluated by modeling their effects on the appropriate demographic stages and/or sub-populations, and then translating these into species- level impacts [10]. One factor that has hindered the use of mechanistic population models for informing conservation is the paucity of reliable demographic data for many rare and endangered species [2,17]. However, improvements in analytical methodology have enabled the development of robust demographic models even for species where data are limited, both by making use of diverse data sources [3,27] and by using hierarchical multispecies analyses to leverage information from data-rich species to inform data-poor species, while accounting for phylogenetic relatedness or life history similarities [16,19].

We developed a generalized meta-Population Viability model (mPVA) for island-breeding seabirds. Our model is based around a stage-structured projection matrix [8], with spatial structure of dispersed breeding colonies incorporated by embedding demographic matrices for semi- discreet sub-populations (generally Islands or Island groups) within a larger meta-matrix structure representing the dynamics of the entire species. We use Bayesian hierarchical methods to parameterize the model using publicly available data contained in the IUCN Red List of Threatened Species version 2018.2 [17], additional data contained in the Threatened Island Biodiversity Database [26], literature-reported values of seabird vital rates, and solicited expert opinion. The hierarchical model structure ensures that, for each species, parameter estimates are informed by data for all species, weighted by taxonomic relatedness and life history similarity, resulting in higher precision of estimates for data-rich taxa and lower precision of estimates for data-deficient taxa. To estimate the demographic impacts of invasive species (that is, to estimate their effects on baseline vital rates), we use published time series data on seabird abundance at islands where invasive species occur and/or where invasive species have been removed. We fit a Bayesian state space model to these time series to estimate the additional hazards associated with invasive species: the hazard function includes covariates for invasive type, nesting type, body size, island size, and number of co-occurring invasive species (allowing for compensatory mortality at islands with >1 invasive species present – see below, “Model Parameterization”).

We use the parameterized mPVA model to simulate population dynamics of threatened and endangered seabirds, with starting abundances initialized using the most recent IUCN red list status reports. Simulations incorporate both environmental stochasticity and appropriate levels of uncertainty in all parameters. Projections are made for all species, including those with limited or negligible monitoring data; however, the level of estimation uncertainty is appropriately higher for these taxa. We summarize results in terms of the proportion of simulations dropping below a quasi- extinction threshold within a 100-year period. We emphasize that our model is not designed to capture the specific dynamics of any one species or conservation threat, and we note that customized models tailored to individual species and their environmental/anthropogenic threats will invariably be more reliable for evaluating localized dynamics. Instead, our generalized model is designed to evaluate and compare conservation benefits (in terms of their effects on relative quasi-extinction likelihood) of various management options across multiple species, using consistent methodology. We focus specifically on 4 management scenarios: (1) invasive species removals, (2) at-sea mortality mitigation; (3) re- introductions to previously-occupied islands; and (4) translocation to potential breeding islands.

## Model structure

The seabird mPVA is a generalizable mathematical structure for projecting the expected abundance over time of threatened seabird species on islands known to support breeding populations. Following general convention, we use a single-sex projection matrix [8] to describe the demographic transitions of independent (non-chick) female sea birds for population *i* in year *t* [1,^11^,^20^,21]. We assume a pre-breeding census, and thus the first tracked age class are juveniles approaching 1-year of age (i.e. chicks born the previous year that have survived both the breeding season and their first winter). To reduce model complexity and number of parameters we collapse year-classes to stages [10], such that individuals are classified by developmental/reproductive status into three life history stages: (1) sub- adults, (2) breeding adults, and (3) non-breeding adults.

We represent the number of individuals in stage *a* in breeding population *i* at year *t* as *n_a,i,t_*, and represent total female abundance for population *i* at year *t* as *N_i,t_* (where *N_i,t_* = Σ*n_a,i,t_*). The three stages are linked demographically in that sub-adults grow and develop to become adults, breeding adults transition to non-breeding adult status (and vice versa) based on behavioral decisions or external constraints, and breeding adults contribute to the sub-adult stage via successful reproduction (i.e., by producing offspring that hatch, fledge and recruit to the sub-adult stage). These demographic transitions are represented mathematically as population projection matrix *A_i_,*

(1) $$A_{i,t}=\left[\begin{array}{ccc} s_{1}\left(1-g\right) & \frac{e}{2}\cdot h\cdot f\cdot s_{0} & 0\\ s_{1}\cdot g & s_{2}\cdot b & s_{3}\cdot b\\ 0 & s_{2}\left(1-b\right) & s_{3}\left(1-b\right) \end{array}\right]$$

where matrix elements are comprised of one or more vital rates including annual survival (*s*), growth transition probability (g), adult annual breeding probability (*b*), average number of eggs produced per breeding pair (*e*), hatching success rate (*h*) and fledging rate of chicks (*f*). Note that *s_0_* represents the survival of fledged chicks for their first winter, while *s_1_* represents sub-adult survival rate, *s_2_* represents breeding adult survival rate, and *s_3_* represents non-breeding adult survival rate. Note that all vital rates are expected to vary stochastically over time (environmental stochasticity), thus the parameterized cell values of *A_i,t_* will vary from year to year (see methods for simulations, below).

To estimate the probability of transitioning from sub-adult to adult stage (g) we use the standard equation for fixed-duration age classes [8]:

(2) $$g=\left(\frac{{\left(s_{1}\;/\;\lambda \right)}^{T}-{\left(s_{1}\;/\;\lambda \right)}^{T-1}}{{\left(s_{1}\;/\;\lambda \right)}^{T}-1}\right)$$

where *T* represents the time from recruitment to the average age of first reproduction (*AFR*) and λ is the annual deterministic growth rate associated with a particular matrix parameterization. Eq. (2) must be solved iteratively: λ is initially set to 1, Eq. (2) and then 1 are solved, λ is re-computed as the dominant eigenvalue of *A_i,t_*, and the calculations repeated until the value of λ stabilizes to 2 decimal places.

Populations of seabirds breeding on oceanic islands are generally embedded within a larger meta- population, consisting of breeding populations at different islands between which there is some level of dispersal and thus demographic connectivity. Multiple breeding populations are accommodated in our model by taking the block diagonal of matrix *Ai,t* across *k* different sub-populations:

(3) $$C_{t}=\left[\begin{array}{cccc} A_{1,t} & \emptyset & \cdots & \emptyset \\ \emptyset & A_{2,t} & \cdots & \emptyset \\ \vdots & \vdots & \ddots & \vdots \\ \emptyset & \emptyset & \cdots & A_{k,t} \end{array}\right]$$

where ∅ represents a 3 × 3 matrix of 0s. To allow for stage-specific dispersal between sub-populations, we first create dispersal matrix *D* to describe dispersal probabilities (*d_a_*) for each life history stage:

(4) $$D=\left[\begin{array}{ccc} d_{1} & 0 & 0\\ 0 & d_{2} & 0\\ 0 & 0 & d_{3} \end{array}\right]$$

We note that *d_a_* represents only the probability that an individual of stage *a* emigrates from source population *i*; it does not specify the recipient population. We next create an inter-population connectivity matrix, *IP*, with non-diagonal elements *p_i,j_* describing the probability that an individual that has emigrated from population *i* will immigrate to population *j* based on the pairwise distances between populations:

(5) $$IP=\left[\begin{array}{cccc} -1 & p_{2,1} & \cdots & p_{k,1}\\ p_{1,2} & -1 & \cdots & p_{k,2}\\ \vdots & \vdots & \ddots & \vdots \\ p_{1,k} & p_{2,k} & \cdots & -1 \end{array}\right]$$

To estimate *p_i,j_* we assumed that distributions of dispersal distances can be reasonably described by exponential distributions, with expected values approximated by the average of literature-reported values of taxa-specific dispersal distances (δ). We used exponential probability density functions to calculate the relative likelihood of dispersal at each pairwise inter-population distance, and then re-scaled these values such that Σ*p_i,j_* = 1 for *i* ≠ *j* . We note that the diagonal of matrix *IP* is fixed at -1 to simplify algebraic calculations (by ensuring that columns of *IP* sum to 1).

To describe annual dynamics of the entire meta-population, we integrate matrices *C, D* and *IP* (following [8]) to obtain meta-population projection matrix *M_t_*

(6) $$M_{t}=\left(IP⊗D\right)\;\times C_{t}+C_{t}$$

In Eq. (6), the Kronecker tensor product of matrices *IP* and *D* describes regional movement transitions for each life stage, while the remaining matrix operations account for demographic transitions of both dispersers and non-dispersers. Annual population dynamics are then computed by taking the product of *M_t_* and the population vector *n_a,i,t_*, using standard methods of matrix multiplication:

(7) $$n_{a,i,t+1}=M_{t}\times n_{a,i,t}$$

In demographic simulation models it is generally important to account for negative density-dependence (the tendency of population growth to decline towards 0 as populations approach environmental carrying capacity or *K*), to avoid unrealistic expectations of unconstrained growth. For threatened seabird demographic models this step is often unnecessary, as current densities are far below historical levels likely to represent K. However, given the time frame of prospective simulations (100 years; see below) and the potential for rapid growth of colonies once critical threats are removed [6], it was necessary to include density-dependence within the mPVA structure. Population regulation in most species occurs due to density-dependent variation in one or more vital rates, although the mechanism and vital rates involved differ by species. For example, Common Guillemots (*Uria aalge*) breeding on the Isle of May, Scotland, experienced density-dependent reduction in breeding probability [9], while Magellanic Penguins (*Spheniscus magellanicus*) experience reductions in fledging success at high densities [25]. In general, both theory and empirical evidence suggest that density-dependent variation is most likely to occur in vital rates with low elasticities [23]; thus, for most seabirds, we would expect population regulation to occur via density-dependent variation in fledging success or juvenile survival, as opposed to adult survival. We therefore modified our model to allow for density-dependent reductions in fledging success (*f*) as populations approach local *K*, using the non-linear function:

(8) $$f_{dd}=f/\left(1+{\left[\frac{N_{i,t}}{K_{i}}\right]}^{\theta }\right)$$

**Fig. 1:**
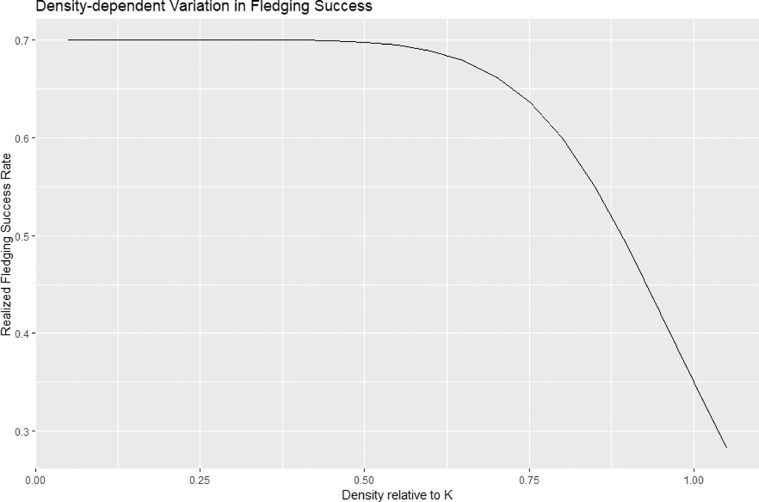
Density-dependent variation in realized Fledging Success rate, as modeled using Eq. (9) (see text for details). At densities below 50% K there is no measurable decrease in baseline fledging success (shown as 0.7 in this example), but as population density increases above 50% K there is an accelerating decrease in fledging success, resulting in zero population growth as the population approaches *K*.

In Eq. (8), *f_dd_* (density-dependent fledging success) varies as a non-linear function of the proportional abundance of a colony relative to *K*. By setting the θ parameter to a value of 5–10, the function results in negligible change in *f_dd_* at densities below 2/3 of K and then accelerating declines in *f_dd_* at higher densities, with a reduction in *f_dd_* of 50% as density approaches *K* (Fig. 1). Another challenge is to define *K* for each seabird/island combination. While *K* has not been defined for most species (let alone specific breeding populations), we can approximate it by multiplying maximum nest density (η) by the potential “Area of Occupancy” (AoO) for the species. Measurements of nest densities are reported in the literature for many species, and the AoO metric is reported by IUCN for many threatened seabirds (IUCN 2018).

## Model parameterization

### Baseline vital rates

The principal benefit of a generalizable seabird mPVA model is that analytical methods are consistent across all seabird species, and thus results (in terms of both quasi-extinction risks and mitigation benefits) can be directly compared across taxa. This beneficial feature also represents a challenge, in that robust estimates of vital rates necessary to parameterize the model have only been published for a fraction of extant seabird species. Moreover, even the literature for data-rich species provides estimates for a sub-set of the total parameters required for the model.

We addressed this challenge by conducting a comprehensive literature search to extract parameter estimates from published reports, and we treat the distribution of reported values as Bayesian priors for our model. Specifically, we reviewed both the primary literature and grey literature (unpublished reports, conference proceedings) to extract all available estimates of vital rate parameters (*s_a_, b*, AFR, *e, h, f, d_a_*, δ and η; Table 1), and their associated standard errors (σ*_v_*), for as many species as possible.

**Table 1:** Seabird vital rate parameters.

***sa***	Adult Survival
***b***	Adult annual breeding probability
**AFR**	Age of first reproduction
***E***	Average number of eggs produced per breeding pair
***H***	Hatching success rate
***F***	Fledging rate of chicks
***da***	Dispersal probability of adults
**δ**	Dispersal distance
**η**	Nest density

**Fig. 2:**
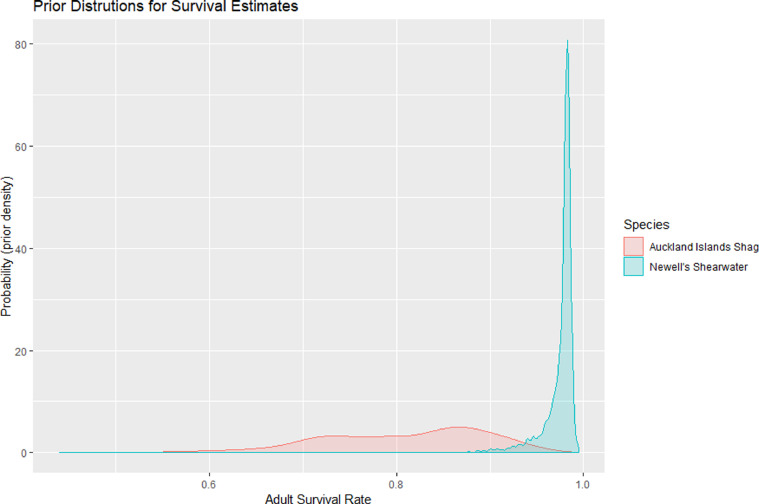
Prior distributions of our estimates for adult survival rate for two species, based on literature searches for published information. The data rich species (blue) has 5 published species-specific estimates, and 107 estimates for the taxonomic Family. The data poor species (red) has no species-specific estimates and only 17 estimates for the taxonomic Family, and this smaller sample size results in a greater degree of uncertainty in the prior distribution.

These were entered into a new table within the Threatened Island Biodiversity Database, augmented by the results of an expert opinion survey mailed to researchers and experts in seabird biology during Fall 2016. The resulting table included at least some estimated values for each parameter, for each seabird family. We next stepped through each seabird species of interest and extracted from the database all parameter estimates available for species from the same taxonomic family as the focal species. We weighted these published estimates in terms of taxonomic relatedness: specifically, we replicated estimates 20x if they were from the same species, 5x if they were from the same genus (but different species) and 1x if they were from a different genus (but same family) from the focal species. We then fit probability density distributions to each sample of estimates, using maximum likelihood estimation (MLE) techniques implemented in R (using library “fitdistr”). In the case of rate parameters (*s_a_, b, h, f* and *d_a_*) we first logit-transformed the sampled estimates and then fit normal distributions to the logit values; for integer parameters (*e* and AFR) we fit Poisson distributions; and for dispersal distances (*δ*) and nest densities (η) we fit log-normal distributions. These MLE-fitted distributions represent the best available prior knowledge about the likely range of values for each parameter value, for each seabird species. For those species or genera that have been well-studied, and for which there are abundant data available, the prior distributions were well defined, whereas for data-poor species the prior distributions were poorly defined, or “vague” (see Fig. 2).

### Estimating carrying capacity

Given the simplifying assumption that the key limiting resource for most seabird breeding populations is appropriate nesting habitat (defined as high quality nest sites at mostly predator-free locations proximal to prey resources), we can derive a rough approximation of carrying capacity (*K*) by multiplying maximum nest density by the total area of appropriate habitat. For many species, information was available from IUCN on the “Area of Occupancy” (AoO), although for most species only a sub-set of AoO represents appropriate nesting habitat. Using those species for which historical data were available on maximum population size (pre-decline) and AoO, we fit a linear model to predict the proportion of AoO used for nesting, with covariates of adult body size and taxonomic family, and used this function to create a scaled value (AoO*) representing total suitable nesting habitat. To estimate *K* at the species level, we multiply range-wide AoO* by mean nest density (η) for each species to get a rough approximation of range-wide *K*. We then partition this total K among the currently occupied breeding locations; however, this step is challenging because equilibrium colony sizes are not equal or random across islands, but rather vary as a function of island size (larger islands generally support larger colonies, but the relationship is non-linear because suitable nesting habitat usually comprises a higher proportion of smaller islands). We therefore used all available survey data to fit a generalized linear mixed-effect model (GLMM) to predict the proportional allocation of seabird abundance as a function of island size and number of breeding colonies:

(9) $$logit\left(\frac{N_{i}}{\sum_{i=1:NI}N_{i}}\right)∼\text{log}\left(Area_{i}\right)+NI+\frac{Area_{i}}{\sum_{i=1:NI}Area_{i}}+\text{log}\left(Area_{i}\right)\times NI+NI\times \frac{Area_{i}}{\sum_{i=1:NI}Area_{i}}+\left(1|Family\right)$$

where *NI* is the number of distinct islands or breeding populations, and taxonomic family is included as a random effect. This model provided a reasonably good fit to the available data and was used to generate expected proportional allocations of range-wide *K* among breeding colonies (subject to the constraint that *Ki* was required to be at least 2x the current estimated abundance). We note that, for most species on most Islands, the resulting estimates of *K* were more than 10x the current abundance estimates.

### Invasive impacts

Multiple studies have documented substantial negative impacts of invasive species on island-breeding seabird colonies (see [18]). However, in most cases the impacts of invasive species are reported in terms of their population-level impacts on abundance or trends, rather than in terms of per- capita effects on specific vital rates. Moreover, these published accounts are generally situation-specific, and thus extrapolating from these case studies to other seabird species and islands is difficult.

Therefore, to provide a consistent and repeatable approach for predicting the effects of invasive species on seabird vital rates, we developed a Baysesian state-space model with which to estimate generalized invasive impacts, incorporating the effects of known co-variates (e.g. class of invasive species, seabird nesting strategy, seabird body size, etc.) while accounting for uncertainty. We used published data on the population trends of seabirds at islands having different suites of invasive species, as well as population trends of seabirds at islands from which invasive species had been removed, to fit this model. To accommodate simultaneous impacts from multiple invasive species (i.e. competing risks), we use a proportional hazards approach to model invasive effects on key vital rates [4,13].

To model invasive impacts, we first assume that the effects of invasive species on breeding seabirds can be described in terms of changes to either or both of two vital rates, fledging success (*f*) and adult survival (*s_2_*). We recognize that other vital rates could also be impacted (hatching success, breeding success, juvenile survival), however given the mPVA matrix structure (Eq. (1)) these effects would be mathematically indistinguishable from effects to *f* or *s_2_*. We next assume that the additional hazards associated with invasive species effects will modify baseline vital rates as follows:

(10) $$f^{\prime }=f^{\text{exp}\left(\gamma_{f}\right)}\;and\;{s_{2}}^{\prime }=s_{2}^{\text{exp}\left(\gamma_{s}\right)}$$

where *γ_f_* and *γ_s_* represent the cumulative log hazard ratio associated with invasive effects on fledging success and adult survival rates, respectively. Note that expressing hazard ratios in log form simplifies calculations and data fitting, as multiple independent hazards are additive in log form [4]. If we define hazard ratio Δ*x* as the proportional change in mortality risk for eggs or nestlings associated with the presence of invasive species *x* (e.g. Δ*x* = 1.1 indicates a 10% increase in mortality risk), and further assume that effects of multiple invasive species are independent and additive, then the cumulative log hazard ratio associated with invasive effects on fledging success (*γf*) would be calculated as the sum of *log*(Δ*x*) for invasive species *x* = 1, 2, …*Xi* (if there are *Xi* invasive species at breeding site *i*). However, because of the brief duration of breeding seasons and concentrated nature of seabird breeding colonies on oceanic islands, it is reasonable to expect that mortality from multiple invasive species is at least partially compensatory rather than purely additive [7,14]. Also, we might expect mortality impacts from invasive species to be more acute on smaller islands, where the potential for refuge from predators is minimal. Accordingly, we compute cumulative log hazards of invasive effects on fledging success at site *i* as:

(11) $$\gamma_{f,i}=\left[\sum_{x=1}^{x=X_{i}}log\left(\Delta_{x}\right)\right]\times {\left(1\;/\;X_{i}\right)}^{\phi }\times {\left(1\;/\;Area_{i}\right)}^{\psi }$$

In Eq. (11), the second term on the right adjusts for compensatory mortality, with parameter *ϕ* determining the degree to which mortality is compensatory (mortality is purely compensatory as *ϕ→*1, purely additive as *ϕ→*0, and 0<*ϕ* <1). The third term on the right of Eq. (11) adjusts for the effect of island size (*Areai* expressed in units of km^2^), such that per-capita impacts decrease with larger Island size when parameter *ψ* > 0. Both *ϕ* and *ψ* are treated as parameters to be fit. The cumulative log hazards associated with invasive effects on adult survival are calculated almost identically to Eq. (11). We note however that the proportional effects of invasive species on adult survival are generally somewhat lower than the effects on chicks and may vary depending on adult size and the type of invasive species (with some invasive species posing no threat to adult seabirds). We therefore replace the nestling hazard ratio Δ*_x_* with an adult hazard ratio, Ω*_x_*, and then estimate cumulative log hazards of invasive effects on adult survival as:

(12) $$\gamma_{s,i}=\left[\sum_{x=1}^{x=X_{i}}log\left(\Omega_{x}\right)\right]\times {\left(1\;/\;X_{i}\right)}^{\phi }\times {\left(1\;/\;Area_{i}\right)}^{\psi }$$

where log(Ω*_x_*) is calculated as:

(13) $$log\left(\Omega_{x}\right)=\zeta_{x}\times \beta_{1}\times log\left(\Delta_{x}\right){\left(1\;/\;AdSz+1\right)}^{\beta_{2}}$$

In Eq. (13), *β_1_* and *β_2_* are fitted parameters that adjust adult hazards relative to chick hazards as a function of adult body size, and *ζ_x_* is a binomial switch variable that determines whether a given invasive species represents a measurable risk to adults, based on published accounts and/or expert opinion (*ζ_x_* = 0 for herbivores and most birds, *ζ_x_* = 1 for carnivores, most omnivores, and rats).

We use Bayesian methods to estimate the scalar parameters *ϕ, ψ, β_1_*, and *β_2_*, and we treat Δ*_x_* as a hierarchical parameter drawn from a normal distribution, Δ*_x_* ∼ ***N*** (Δ,*σ* Δ), where Δ and σ_Δ_ are additional parameters to be fit. To limit the number of fitted parameters we did not estimate unique values of Δ*_x_* for each combination of invasive species and seabird, but rather for each combination of nesting type (arboreal, burrow, cliff, crevice, crevice/burrow, surface) and 5 categories of invasive species (bird, carnivore, herbivore, omnivore, rat). Our observed data were time series of survey counts (*O_i,t_*) at islands supporting different suites of invasive species, as well as survey counts at islands where invasive species had been present but then were removed [6]. The latent (unobserved) variable was the true abundance of each seabird species at each island (*N_i,t_*), assumed to be affected by the presence of (or removal of) invasive species. For each available survey estimate, *O_i,t_* was assumed to be drawn from a Poisson distribution with mean *N_i,t_*. The dynamics of *N_i,t_* were calculated using standard matrix multiplication methods, with projection matrices constructed and parameterized according to Eqs. (1), (2), (10)–(13) (note that for this analysis we ignored density-dependence and inter-island dispersal). Priors for baseline vital rates were set according to the methods described above, and we used uninformative priors for *N_i,1_* and for the parameters that determined invasive species effects (*ϕ, ψ, β_1_, β_2_*, Δ and σ_Δ_).

The model was coded in R and JAGS (Just Another Gibbs Sampler) and solved using Markov Chain Monte Carlo methods to find the values of the parameters most likely to result in the observed data. We ran 20 parallel chains for a burn-in period of 5000 replications and then saved a total of 10,000 samples, using these to describe the posterior distributions for invasive species effects parameters.

### Initializing meta-population and incorporating information on current trends

Before running simulations of the mPVA for a seabird species of interest, the model was initialized with starting abundances at each breeding island. For some threatened species, estimates of the total number of adult birds or the number of breeding pairs are available for each occupied island. These data were obtained through searches of primary and grey literature as well as from BirdLife International species factsheets (http://datazone.birdlife.org/species/spcreferences). When island specific estimates of total birds are available, we simply divide the value in half (to obtain the estimated total number of female birds) and then multiply by the stationary stage distribution (SSD) associated with the parameterized matrix model [8], in order to create the initial population vector *n_a,i,1_*. When island specific estimates of breeding pairs are available, we use this value to estimate *n_2,i,1_*, and then calculated scaled estimates of sub-adults and non-breeding adults using the SSD. For many other species, estimates of abundance are only available for the entire population, and so the estimate must be partitioned among breeding sites/islands. We accomplish this using the fitted proportional allocation function (Eq. (9)) to partition the total number of female birds among breeding colonies, accounting for prediction uncertainty and sampling error as described above for “Estimating Carrying Capacity”. The total number at each island is then divided among stages according to the SSD.

For most threatened seabird species of interest, the IUCN Red List also includes information on current population trends for each species. We used this information to update the prior distributions of parameter estimates for each species, thereby ensuring that the mPVA model simulations were consistent with the best available information on current trends. To accomplish this, we created *a priori* quantitative definitions for the expected values of λ (annual growth rates) corresponding to the qualitative descriptions of status/trends in the IUCN red list (Increasing, Stable, Decreasing). Based on reported quantitative trend values available for a sub-set of species, we assumed modal lambda values of 1.02, 1.00 and 0.98 for Increasing, Stable and Decreasing, respectively; however, recognizing the uncertainty associated with the qualitative status designations we also allowed for a distribution of uncertainty around each modal value. Specifically, for each classification we assigned relative weights (*w*_λ_, where Σ *w*_λ_ = 100) corresponding to our expectations about the likelihood of each potential value of lambda for a given status, assuming a distribution of possible log(lambda) values with standard deviation = 0.03 Table 2). Using these weights as sample sizes, we created a vector of 100 “observed trend values” for each species/island combination, corresponding to the reported trends in the IUCN Red List. We then created a Bayesian model (coded using JAGS software) to estimate posterior distributions for all model parameters, given the set of prior expectations (i.e. the MLE-fitted distributions for baseline vital rates and posterior distributions for invasive threat function parameters) and the observed trend values. As described above (see “*Invasive Impacts*”), Eqs. (1), ((2) and (10)–(13) were used to calculate expected dynamics of the latent variable (*Ni,t*) and thus the mean annual growth rate ($\hat{\lambda }$) associated with a given set of parameter values; the observed trend values were assumed to be drawn from a log-normal distribution with mean of $\hat{\lambda }$ and standard error σ_λ_ (itself a fitted parameter). We saved 5000 samples from the Bayesian posterior distributions for each parameter, for each species/island pair, and used these to parameterize model simulations (see below).

**Table 2:** Weights used to define "observed values" for annual trend (λ) associated with qualitative descriptions of trends in IUCN status reports. The distribution of λ weights provides an approximation of the uncertainty associated with quantitative population trends.

*Lambda*	Increasing	Stable	Decreasing	Unknown
*0.9*	0	0	0	1
*0.91*	0	0	1	1
*0.92*	0	0	2	2
*0.93*	0	1	3	3
*0.94*	0	2	6	3
*0.95*	1	3	8	5
*0.96*	2	5	11	6
*0.97*	3	8	12	7
*0.98*	6	11	13	9
*0.99*	8	13	12	10
*1*	11	14	11	10
*1.01*	12	13	8	10
*1.02*	13	11	6	9
*1.03*	12	8	4	7
*1.04*	11	5	2	5
*1.05*	8	3	1	4
*1.06*	6	2	0	3
*1.07*	4	1	0	2
*1.08*	2	0	0	1
*1.09*	1	0	0	1
*1.1*	0	0	0	1
*Tally Wts*	100	100	100	100

## Running Simulations to assess and compare relative risk

To evaluate the relative degree of extinction risk for seabird species, and to examine and compare the potential benefits of alternative management actions, we conducted forward simulations using the mPVA model. After drawing parameter values randomly from their appropriate uncertainty distributions, we simulated 100 years of population dynamics for each species, with the effects of year- to year variation in environmental conditions (environmental stochasticity) represented by adding a zero-centered random normal term to the logit-transformed vital rates. We assumed that annual deviations from average survival were perfectly correlated across stages but with the magnitude of variance (σ*_e_*) allowed to differ by stage: for species having reliable data on the magnitude of annual variance in vital rates we used these data to set σ*_e_*, otherwise we used default values of σ*_e_* = 1 for fledging survival rates and σ*_e_* = 0.5 for all other stages. We next adjusted environmental stochasticity to incorporate temporal and spatial autocorrelation: we used the “filter” function in R (which uses a Fast Fourier Transform to convolve a time series of random values to achieve a specified autocorrelation) to transform the annual deviations, setting the average first-order correlation across years to *R* = 0.67. We used the inverse matrix of between-colony distances to parameterize spatial autocorrelation, scaled such that two colonies 100km apart would have correlated annual deviations with *R*=0.9, while two colonies 1000km apart would have correlated annual deviations with *R* = 0.5. Finally, when the population abundance at a breeding colony dropped below 100, we adjusted the calculation of annual demographic transitions to allow for demographic stochasticity: specifically, adjusted survival parameters were drawn from a beta distribution with mean equal to the expected value and variance equal to (*p* * *q*)/*n* (where *p* is the mean expected value, *q* = 1 – p, and *n* is the number of individuals in the stage experiencing the survival rate).

**Fig. 3:**
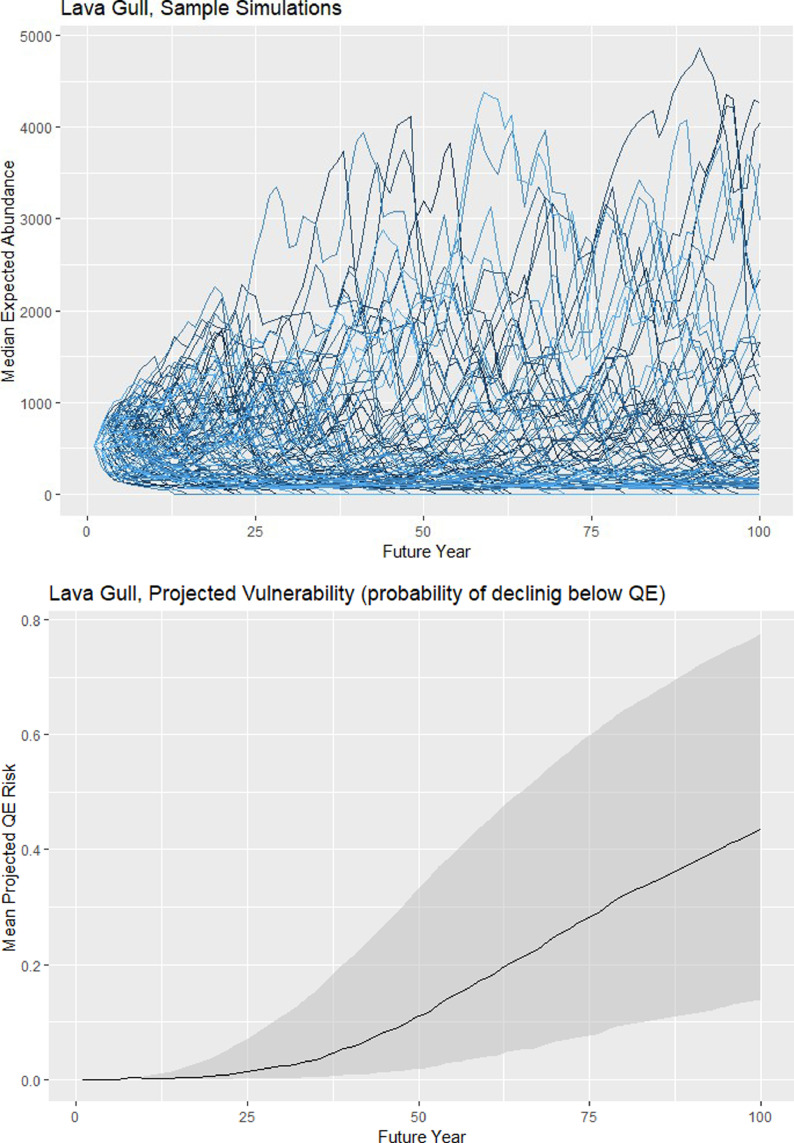
TOP PANEL: Sample population abundance trajectories over a 100-year period as projected by simulations of the mPVA model run for a sample species (Lava Gull). Each line shows a single 100-year simulation, with variation between lines representing uncertainty due to sampling error and environmental stochasticity. Simulation runs that drop below the QE threshold (50 females) are assumed to go extinct. BOTTOM PANEL: Projected vulnerability for sample species plotted over time, where projected QE risk is defined as the proportion of simulations that decline below the QE threshold. Solid line shows mean values and grey shaded band indicates the inter-quartile range for all simulations.

We iterated the population dynamic simulations many times so that the distribution of results could be used to describe the uncertainty associated with model projections (Fig. 3). We ran simulations for the “default scenario”, corresponding to the current species distribution, abundance and array of threats, and under “alternative scenarios” corresponding to various management actions (invasive species removals, reductions of by-catch or other at-sea mortality, translocations, or re-introductions). As a metric of comparison, we use quasi-extinction probability (QEP), defined as the relative likelihood that model-projected abundance would drop below a quasi- extinction threshold within a 100-year period. Quasi-extinction thresholds (QE) are often used in PVA models as a surrogate for absolute extinctions [5,24], describing the point at which abundance is so low that true extinction risk due to natural catastrophes, demographic stochasticity or loss of genetic diversity becomes unacceptably high. There are no universally accepted definitions of QE (but see [15]): values of *N*=500 have been suggested based on genetic considerations, but lower values (100 or 50) may be more appropriate for large/rare species. We set QE to 50 females (100 individuals) for species with an initial population exceeding 200 breeding pairs, or to 10 females for those species with an initial population less than or equal to 200 breeding pairs.

We used two hierarchical levels of replication for model simulations. An outer loop was used to account for parameter uncertainty, whereby for each of 100 replications (*NS_1_* = 100) we made random draws of all parameter values from their respective posterior distributions (as described in the sections above). For each outer loop replication, we conducted an inner loop of 100 iterations (*NS_2_* = 100) of the 100-year simulation, to account for uncertainty associated with environmental and demographic stochasticity and sampling error. The distribution of simulation outcomes from the inner loop was used to calculate a point estimate of projected abundance (*N_proj_*) and QEP (proportion of simulations dropping below QE) for each iteration of the outer loop (Fig. 3). We then calculated the median, standard error and inter-quartile range of *N_proj_* and QEP distributions across outer loop replicates. These metrics were used to evaluate relative risk for seabird species and to compare the efficacy of alternative management scenarios. We emphasize that QEP values are intended as relative measurements of risk only, and not intended to be accurate predictions of extinction risk [22,24].

## Declaration of Competing Interest

The authors declare that they have no known competing financial interests or personal relationships that could have appeared to influence the work reported in this paper.
